# Molecular Imaging in Targeted Therapeutics

**DOI:** 10.1155/2018/3236829

**Published:** 2018-09-05

**Authors:** Yuebing Wang, Zhen Cheng, Shuang Liu, Guoqiang Shao

**Affiliations:** ^1^Department of Biochemistry, School of Medicine, Nankai University, Tianjin 300071, China; ^2^Molecular Imaging Program at Stanford (MIPS), Canary Center at Stanford for Cancer Early Detection, Department of Radiology and Bio-X Program, School of Medicine, Stanford University, Stanford, CA 94305, USA; ^3^School of Health Sciences, Purdue University, 550 Stadium Mall Drive, West Lafayette, IN 47907, USA; ^4^Department of Nuclear Medicine, Nanjing First Hospital, Nanjing Medical University, Nanjing 210006, China

With the rapid development of precision medicine, targeted therapy of tumors has made great advances. However, tumors of the same type were found to harbor different molecular alterations such as specific receptor, transporter, and cell expression levels before and even during the targeted therapy. Molecular imaging allows mapping of disease markers *in vivo* and understanding of disease biology. It promotes targeted therapy by therapeutic effect prediction, early response evaluation, and therapeutic regimen determination in preclinical and clinical settings. Molecular imaging technologies are being recognized as one of the most valuable tools in the field of precision medicine, which will make individual treatment decisions for better patient outcome care.

In this special issue, we focus on the recent advances of molecular imaging for targeted treatment strategies in precision medicine. Two review articles and nine original research articles are published in this special issue. The topics included synthesized targeting probes: PET, SPECT, MR, ultrasound, fluorescence, photoacoustics, and multimodal imaging.

L. P. Yao et al. reviewed the features of even blues and their potential applications in biomedicine. They pointed out that radiolabeled even blue and its derivatives will play an important role in clinical imaging of tumor lesions, evaluation of lymphatic disorders, and development of long-acting therapeutics. In the other review paper, A. C. Dupont et al. summarized the current inputs of PET in the assessment of therapeutic effectiveness in neurodegenerative diseases connected by common pathophysiological mechanisms. In addition, they discussed the opportunities for PET imaging to drive more personalized neuroprotective and therapeutic strategies.

Smart targeting probes were designed by the researchers in this special issue. Y. Y. Zhang et al. developed a smart nanoprobe named [^131^I] GNR-PEG-cRGD, which can be used for angiogenesis-targeted SPECT/CT imaging. This nanoprobe possesses a remarkable capacity for highly efficient photothermal conversion in the near-infrared region, suggesting its potential as a multifunctional theragnostic agent. Z. Q. Hu et al. designed NPs-SHP2 nanoparticles, which had high specificity to thyroid tumors *in vitro* and *in vivo*. Moreover, NPs-SHP2 could be activated by LIFU irradiation to enhance ultrasound molecular imaging in thyroid cancer model. In the study by X. B. Ma et al., GO-AuNS-DOTA-Gd was prepared and its physicochemical properties indicated that this probe could act as an efficient photosensitizer for photothermal therapy. Additionally, GO-AuNS-DOTA-Gd can be used for precisely delineating the tumor margin from normal tissues based on multimodality molecular imaging.

A new tracer [^18^F] AmBF_3_-TEG-ES was prepared by H. B. Huang et al. They showed that [^18^F] AmBF_3_-TEG-ES would be a potential PET imaging agent for the diagnosis of estrogen-dependent tumors. Similarly, X. Q. Du et al. investigated the differential diagnostic value of ^18^F-Alfatide II PET/CT between tuberculosis and lung cancer patients. Moreover, they analyzed the angiogenesis in sarcoidosis and chronic inflammations.

R. Nishii et al. also focus on PET imaging. They demonstrated that MeAIB-PET could provide better assessments for detecting malignant type brain tumors. In a differential diagnosis between low-grade and high-grade astrocytoma, MeAIB-PET is a useful diagnostic imaging tool, especially in evaluations using the T/N ratio.

Y. J. Liu et al. observed that positive neural correlations of respiratory amplitude were shown in anterior lobe and insula, while they were negative in prefrontal cortex and sensorimotor areas. Their findings revealed the involvement of cognitive, executive control, and sensorimotor processing in hypnosis for respiratory control.

G. Q. Shao et al. published two articles in this special issue. In one of the papers, they synthesized a novel ^32^P-CP-PLGA seeds which can control the release of entrapped ^32^P-CP particles. Their results demonstrated that ^32^P-CP-PLGA seeds are very promising for glioma brachytherapy, and ^68^Ga-3PRGD2 imaging shows great potential for early response evaluation of ^32^P-CP-PLGA seeds brachytherapy. In the other paper, they demonstrated that the ^68^Ga-PSMA-11 PET-CT imaging could invasively evaluate PSMA expression during PCa progression and tumor growth with % ID/cm^3^ (based on functional volume) as an important index. Low-dose 2-PMPA preadministration might be a choice to decrease kidney uptake of ^68^Ga-PSMA-11.

